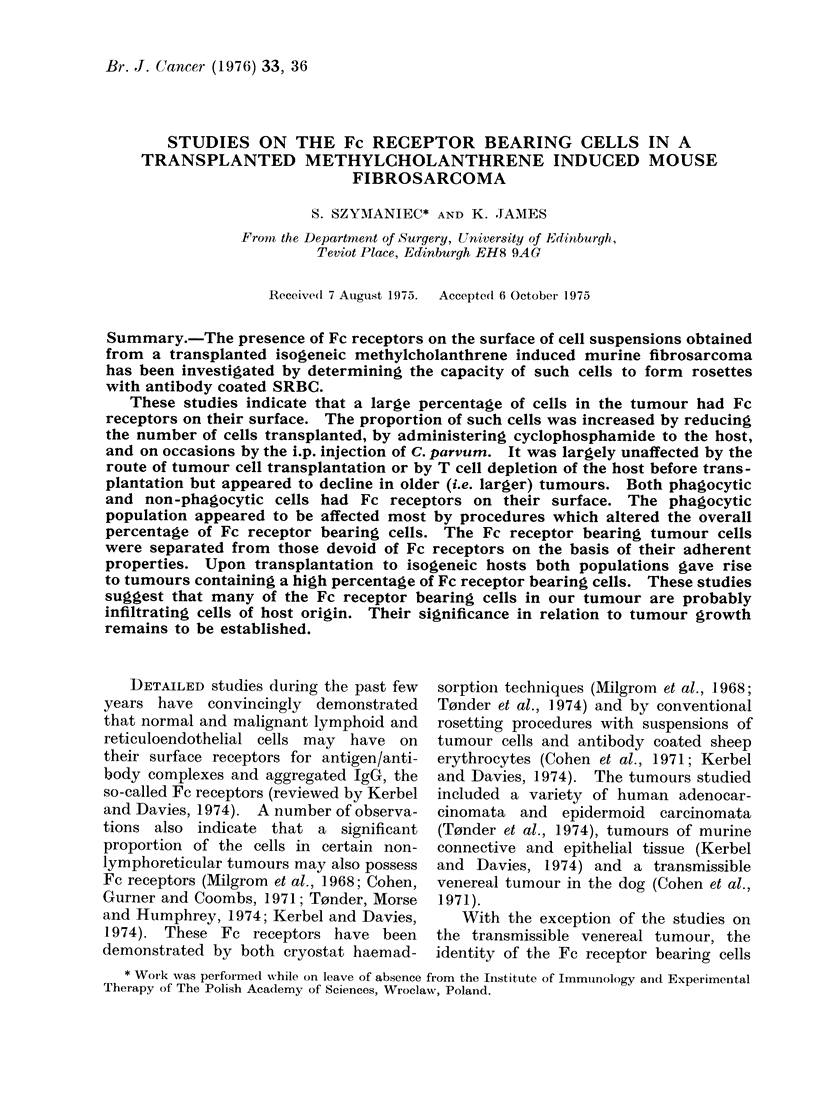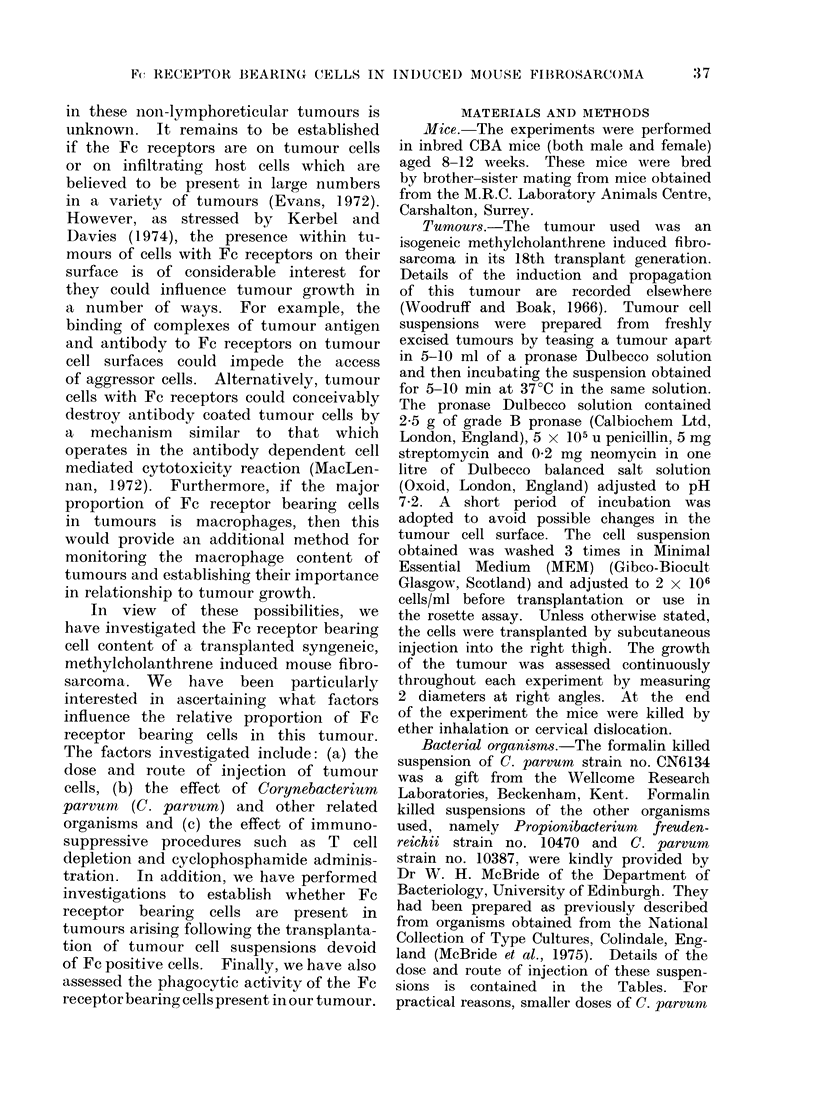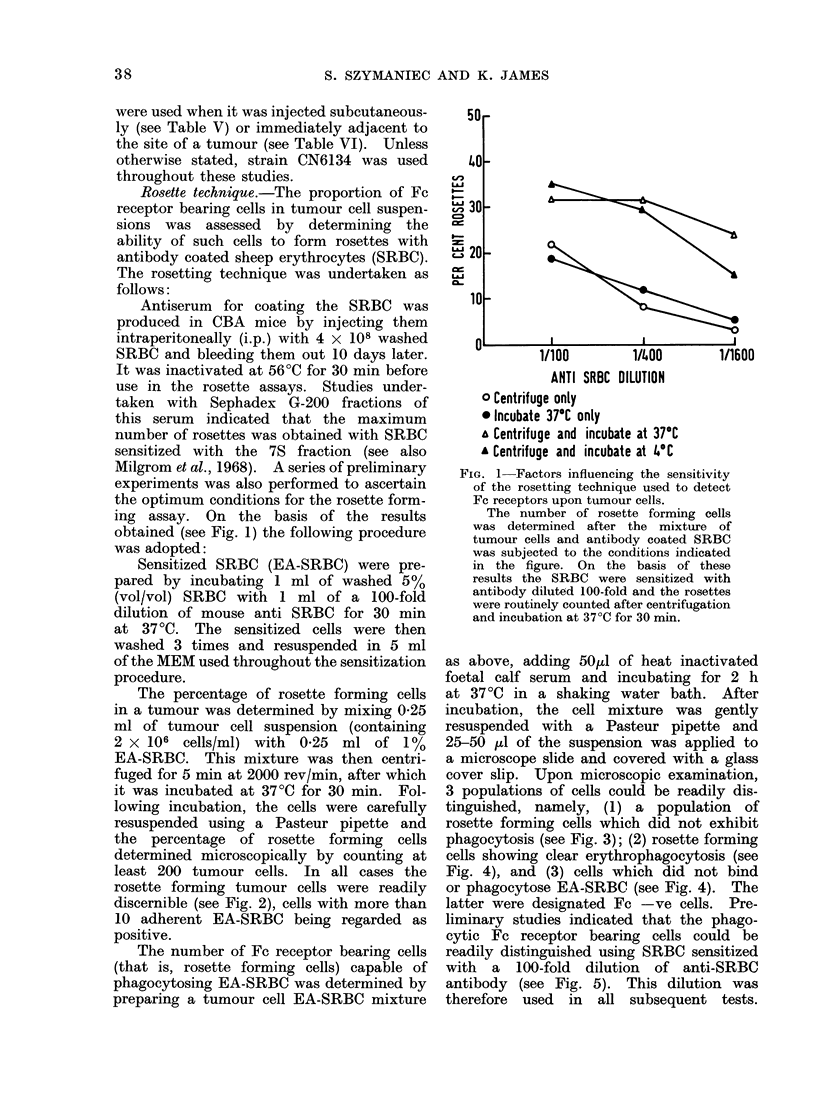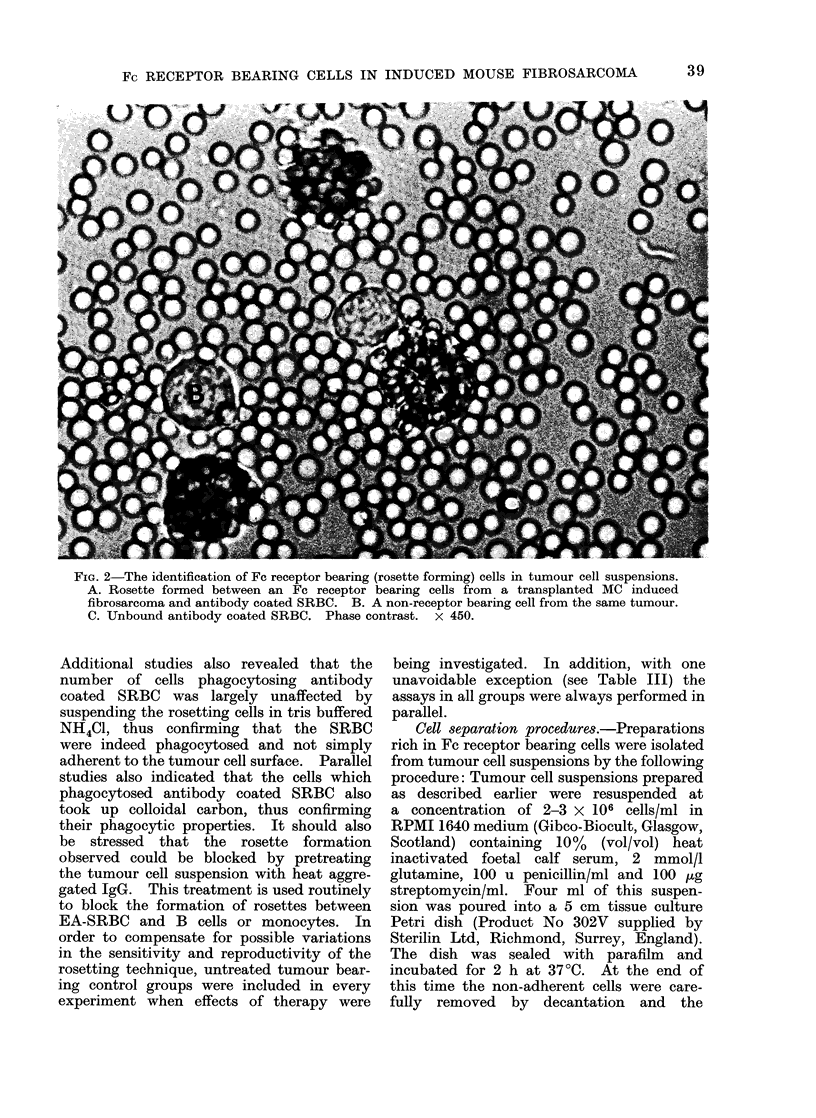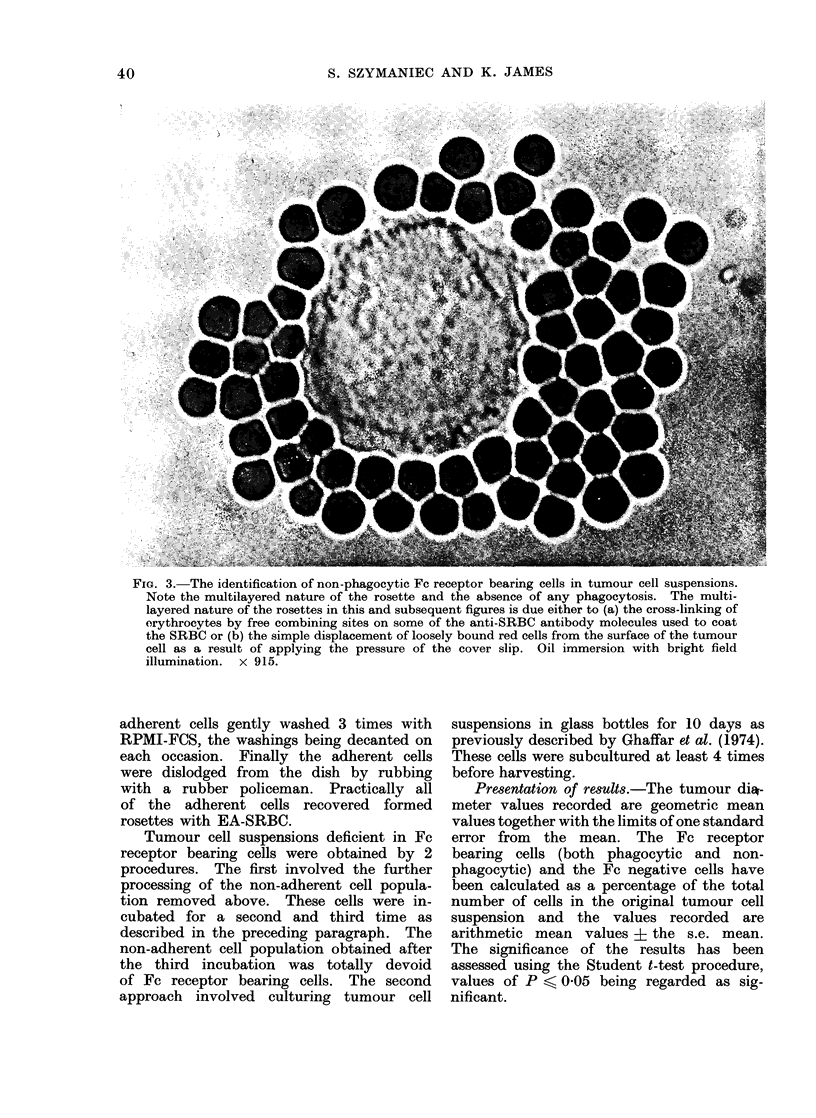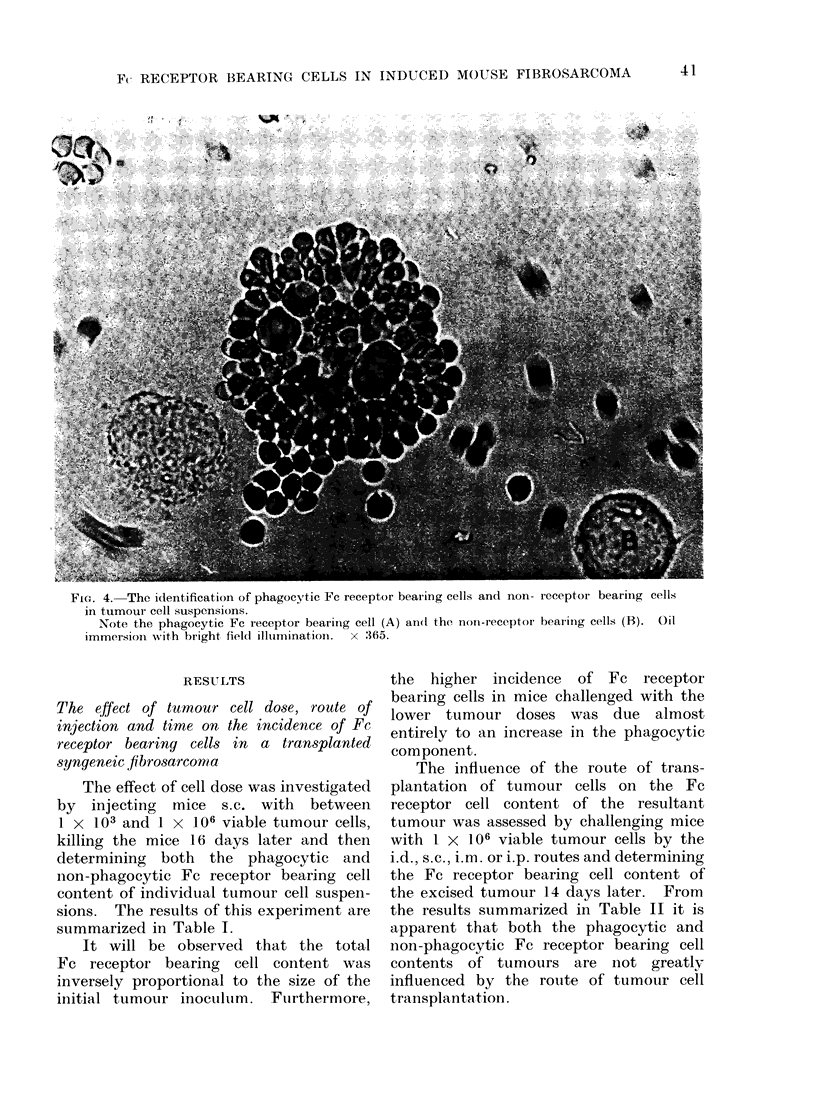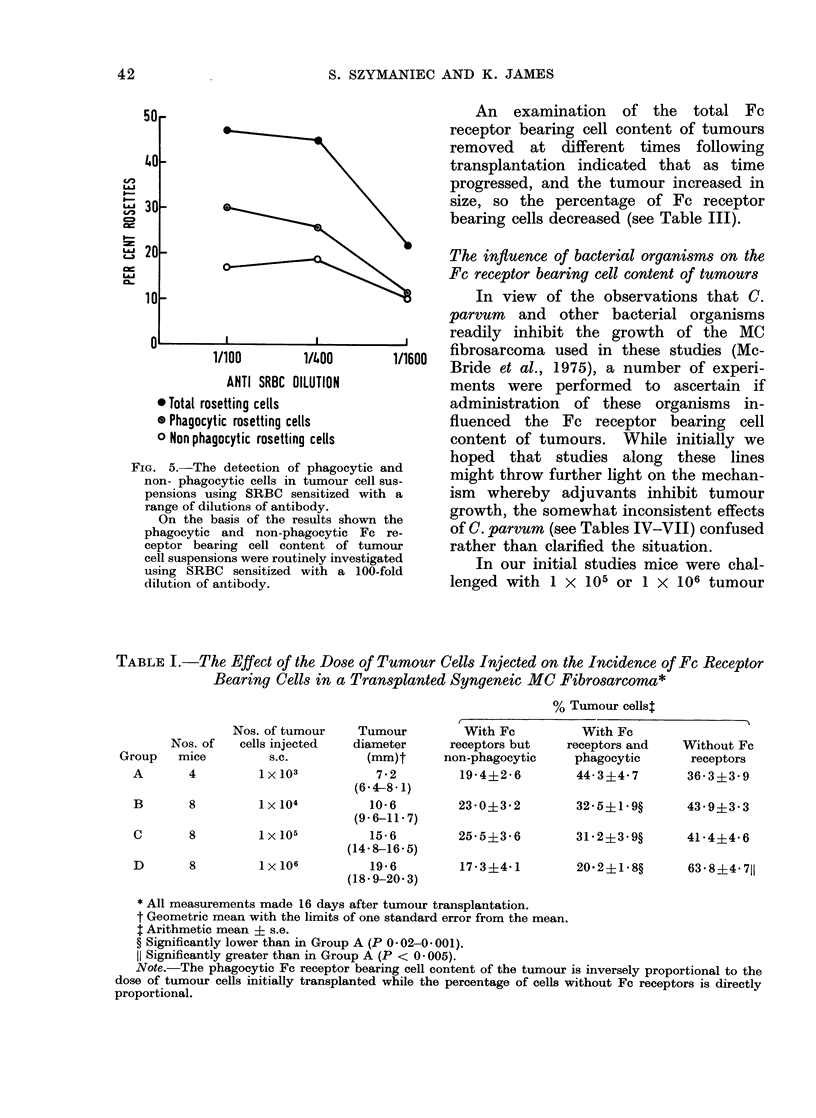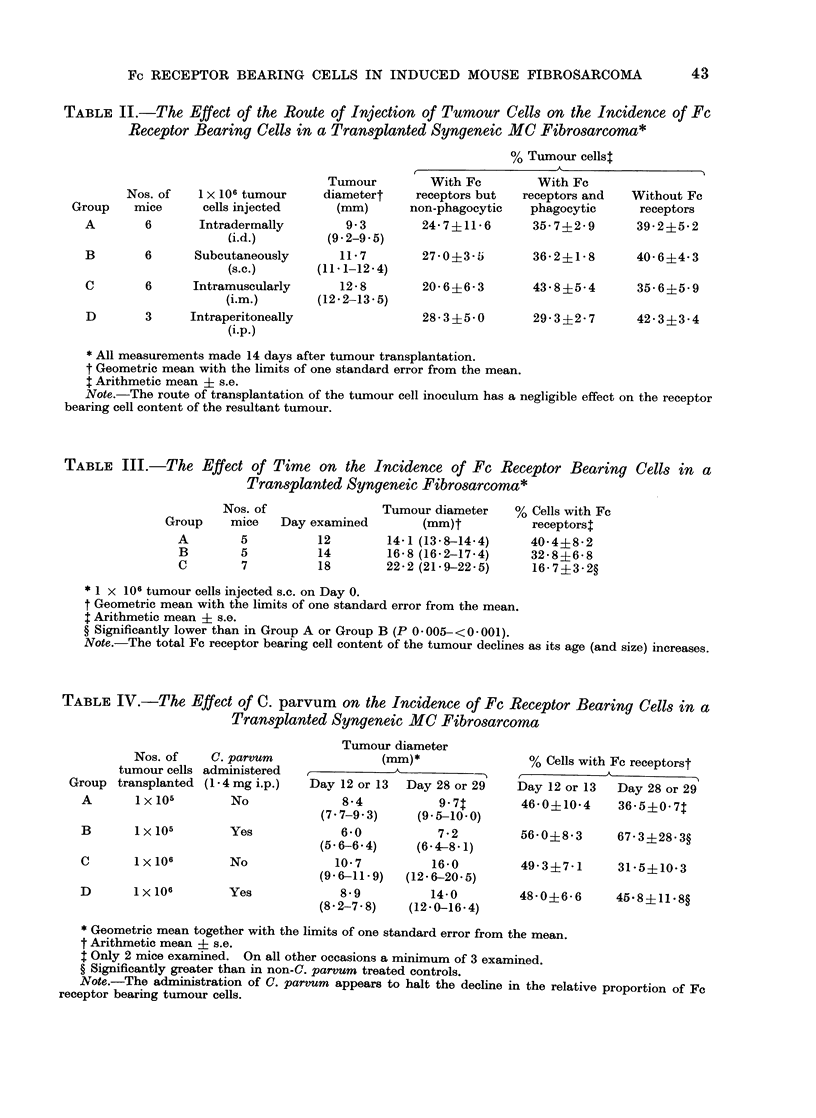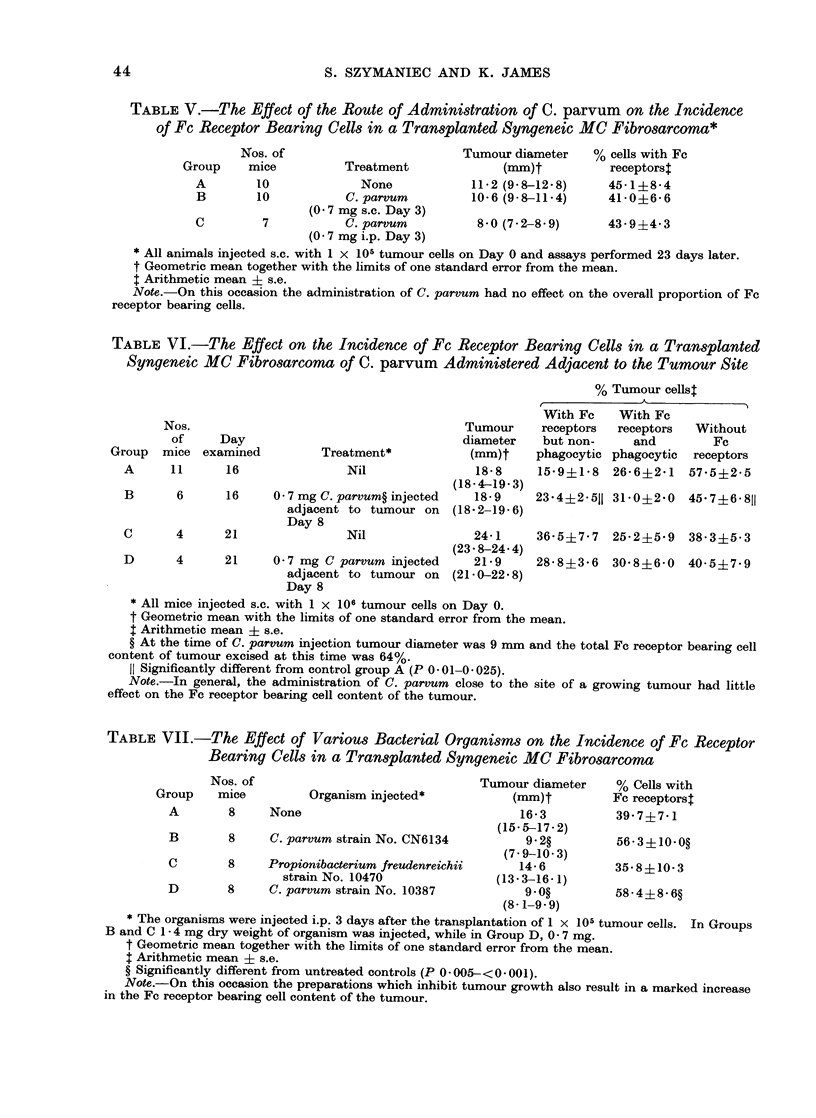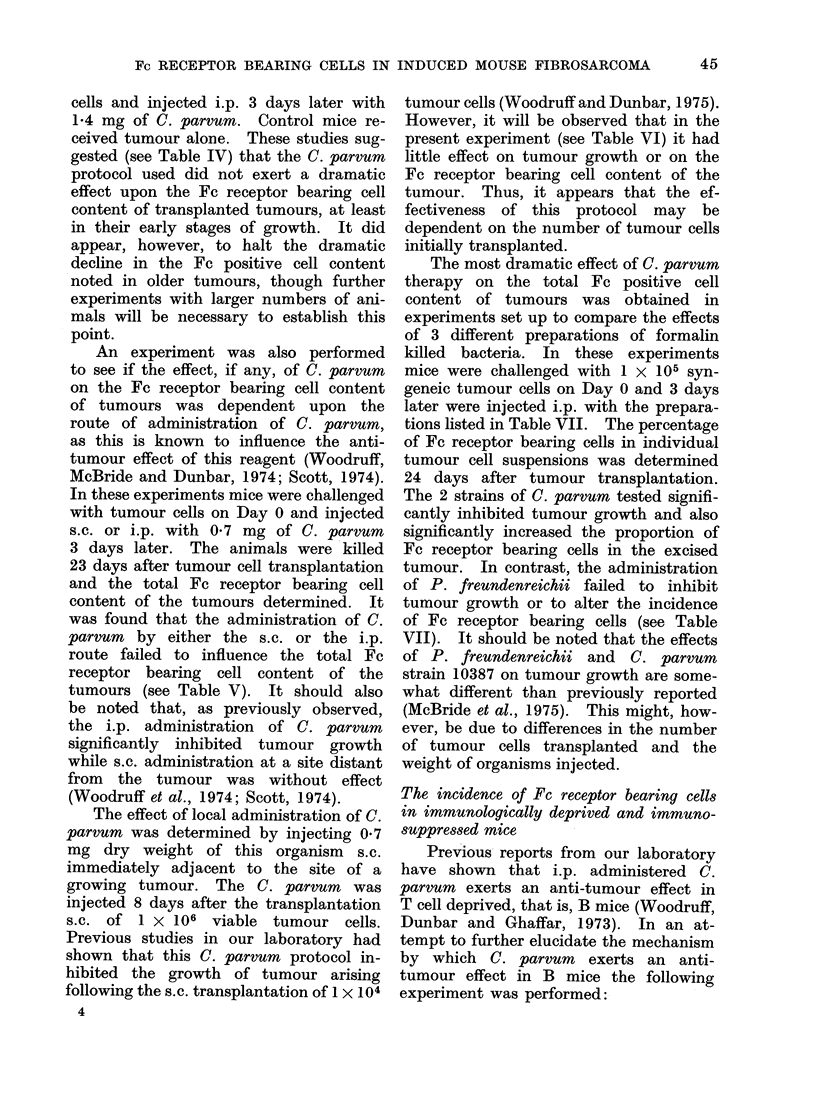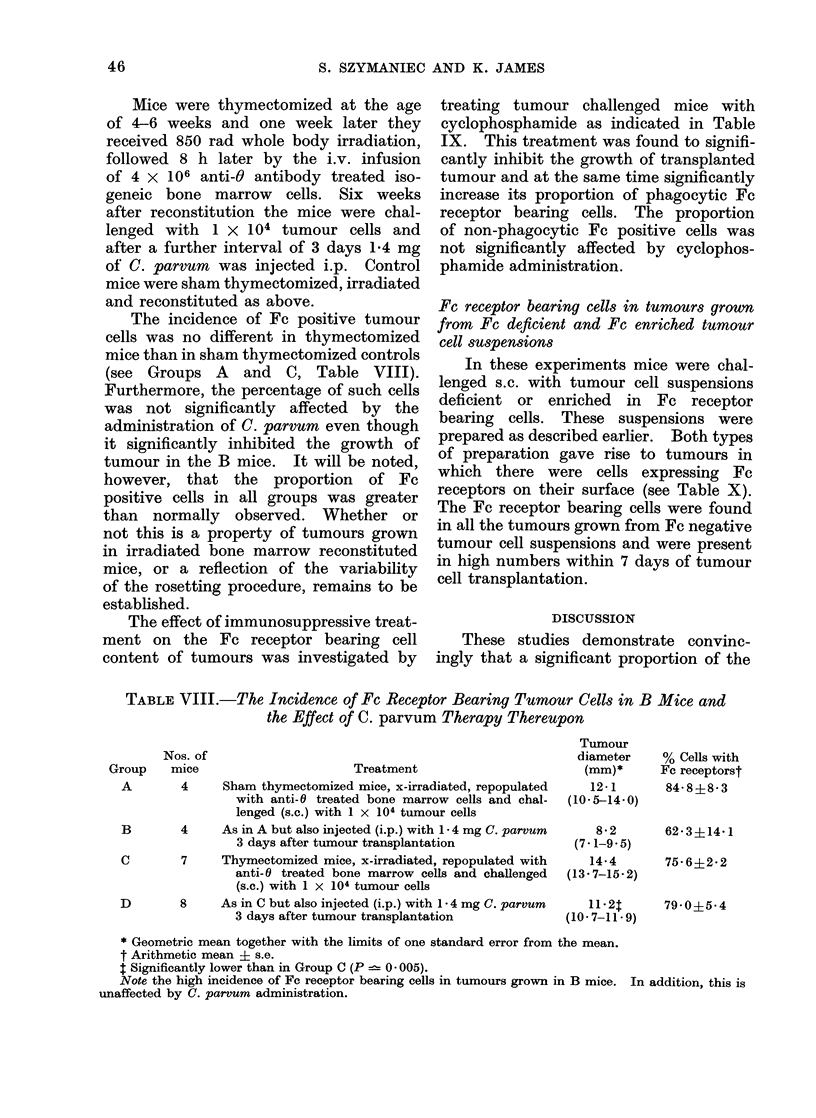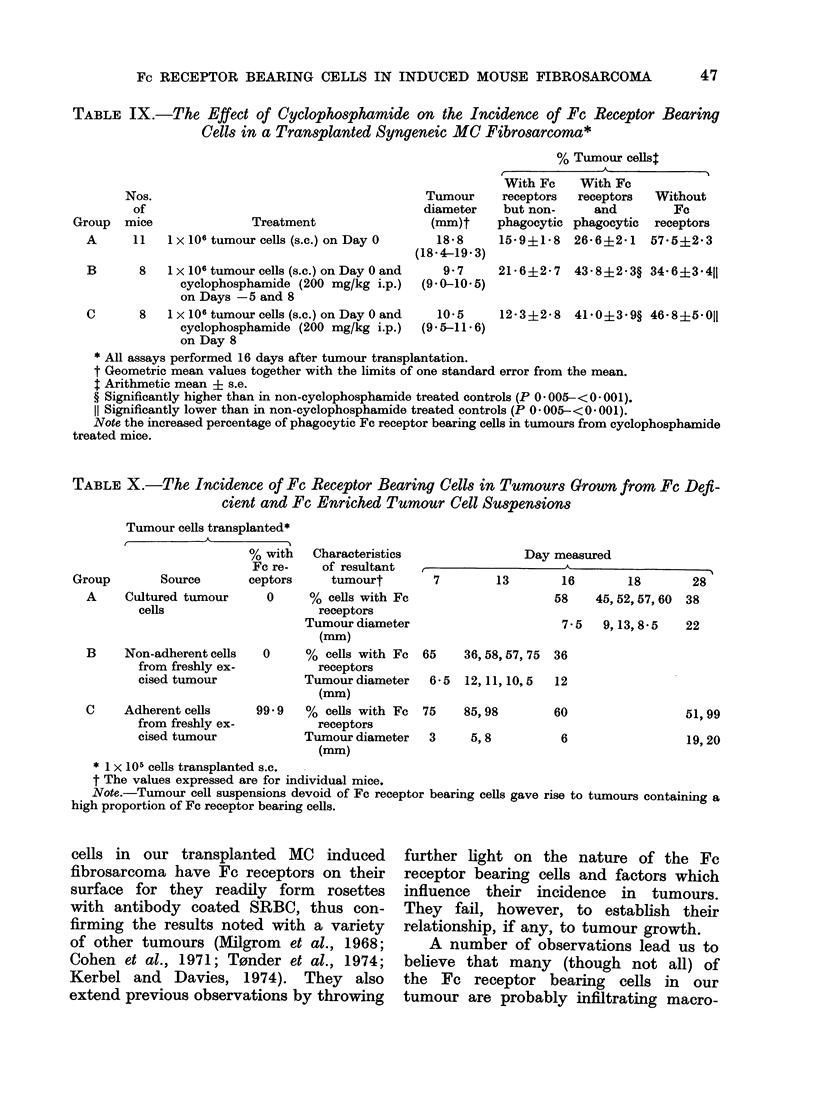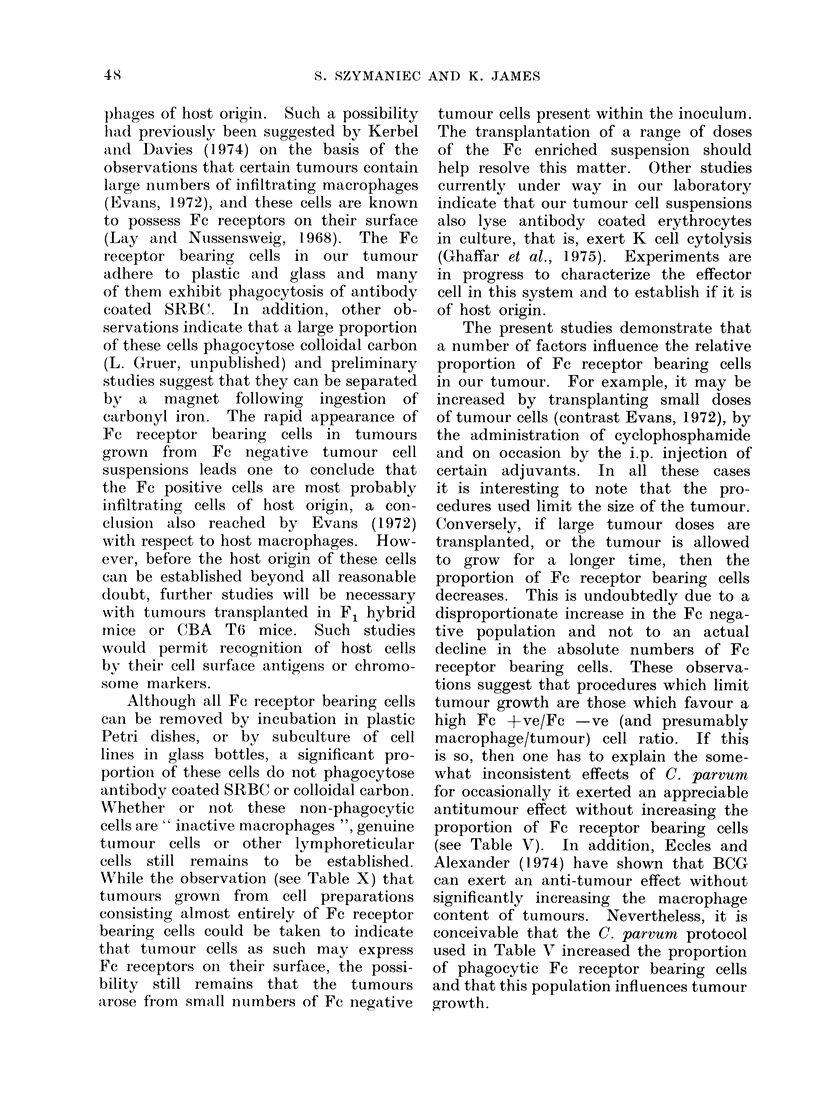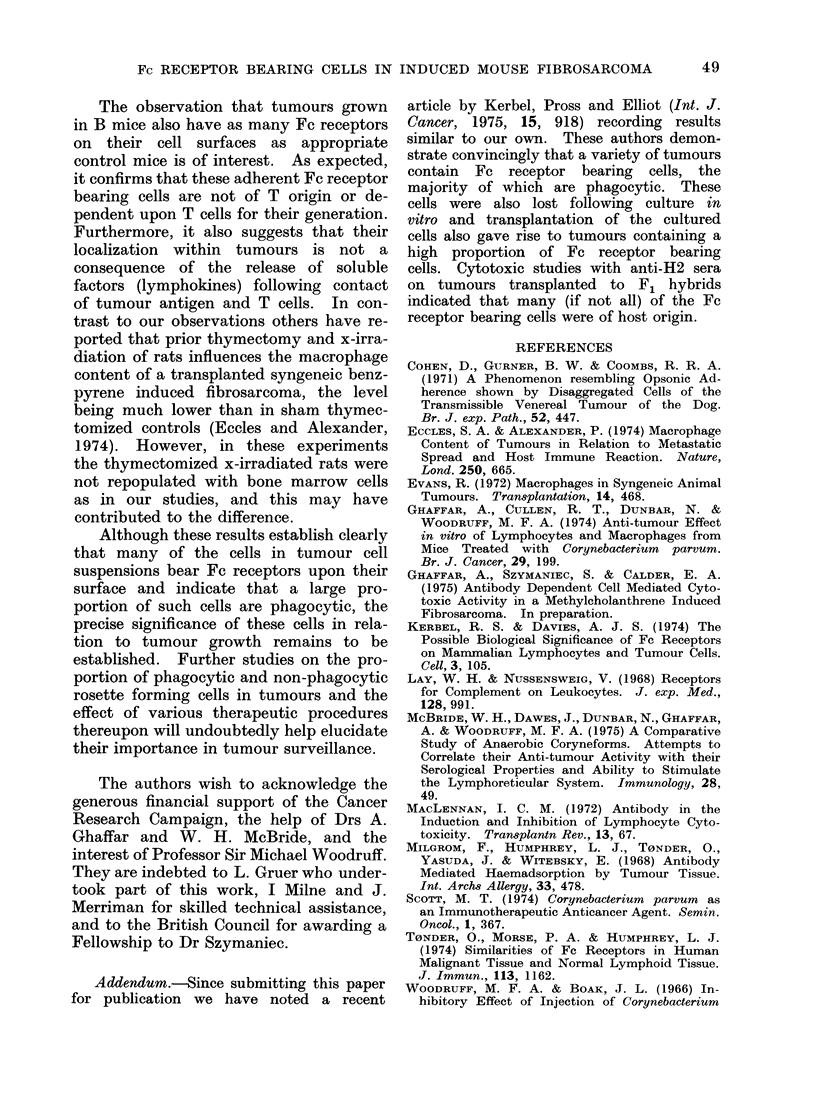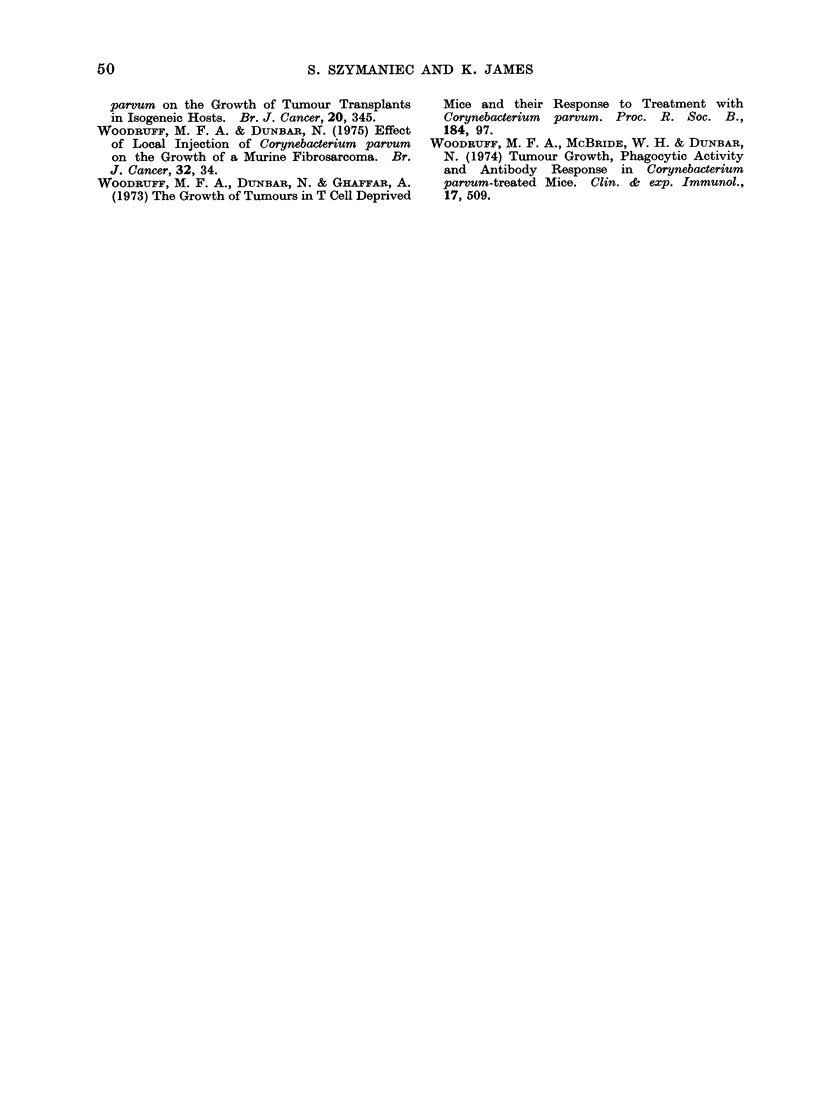# Studies on the Fc receptor bearing cells in a transplanted methylcholanthrene induced mouse fibrosarcoma.

**DOI:** 10.1038/bjc.1976.5

**Published:** 1976-01

**Authors:** S. Szymaniec, K. James

## Abstract

**Images:**


*Br. J. Cancer* (1976) **33**, 36

### STUDIES ON THE Fc RECEPTOR BEARING CELLS IN A TRANSPLANTED METHYLCHOLANTHRENE INDUCED MOUSE FIBROSARCOMA

S. SZYMANIEC* AND K. JAMES

*From the Department of Surgery, University of Edinburgh, Teviot Place, Edinburgh EH8 9AG*

Received 7 August 1975. Accepted 6 October 1975

**Summary.—The presence of Fc receptors on the surface of cell suspensions obtained from a transplanted isogeneic methylcholanthrene induced murine fibrosarcoma has been investigated by determining the capacity of such cells to form rosettes with antibody coated SRBC.**

**These studies indicate that a large percentage of cells in the tumour had Fc receptors on their surface. The proportion of such cells was increased by reducing the number of cells transplanted, by administering cyclophosphamide to the host, and on occasions by the i.p. injection of *C. parvum*. It was largely unaffected by the route of tumour cell transplantation or by T cell depletion of the host before transplantation but appeared to decline in older (*i.e.* larger) tumours. Both phagocytic and non-phagocytic cells had Fc receptors on their surface. The phagocytic population appeared to be affected most by procedures which altered the overall percentage of Fc receptor bearing cells. The Fc receptor bearing tumour cells were separated from those devoid of Fc receptors on the basis of their adherent properties. Upon transplantation to isogeneic hosts both populations gave rise to tumours containing a high percentage of Fc receptor bearing cells. These studies suggest that many of the Fc receptor bearing cells in our tumour are probably infiltrating cells of host origin. Their significance in relation to tumour growth remains to be established.**

DETAILED studies during the past few years have convincingly demonstrated that normal and malignant lymphoid and reticuloendothelial cells may have on their surface receptors for antigen/antibody complexes and aggregated IgG, the so-called Fc receptors (reviewed by Kerbel and Davies, 1974). A number of observations also indicate that a significant proportion of the cells in certain non-lymphoreticular tumours may also possess Fc receptors (Milgrom *et al.*, 1968; Cohen, Gurner and Coombs, 1971; Tønder, Morse and Humphrey, 1974; Kerbel and Davies, 1974). These Fc receptors have been demonstrated by both cryostat haemadsorption techniques (Milgrom *et al.*, 1968; Tønder *et al.*, 1974) and by conventional rosetting procedures with suspensions of tumour cells and antibody coated sheep erythrocytes (Cohen *et al.*, 1971; Kerbel and Davies, 1974). The tumours studied included a variety of human adenocarcinomata and epidermoid carcinomata (Tønder *et al.*, 1974), tumours of murine connective and epithelial tissue (Kerbel and Davies, 1974) and a transmissible venereal tumour in the dog (Cohen *et al.*, 1971).

With the exception of the studies on the transmissible venereal tumour, the identity of the Fc receptor bearing cells

* Work was performed while on leave of absence from the Institute of Immunology and Experimental Therapy of The Polish Academy of Sciences, Wroclaw, Poland.

in these non-lymphoreticular tumours is unknown. It remains to be established if the Fc receptors are on tumour cells or on infiltrating host cells which are believed to be present in large numbers in a variety of tumours (Evans, 1972). However, as stressed by Kerbel and Davies (1974), the presence within tumours of cells with Fc receptors on their surface is of considerable interest for they could influence tumour growth in a number of ways. For example, the binding of complexes of tumour antigen and antibody to Fc receptors on tumour cell surfaces could impede the access of aggressor cells. Alternatively, tumour cells with Fc receptors could conceivably destroy antibody coated tumour cells by a mechanism similar to that which operates in the antibody dependent cell mediated cytotoxicity reaction (MacLennan, 1972). Furthermore, if the major proportion of Fc receptor bearing cells in tumours is macrophages, then this would provide an additional method for monitoring the macrophage content of tumours and establishing their importance in relationship to tumour growth.

In view of these possibilities, we have investigated the Fc receptor bearing cell content of a transplanted syngeneic, methylcholanthrene induced mouse fibrosarcoma. We have been particularly interested in ascertaining what factors influence the relative proportion of Fc receptor bearing cells in this tumour. The factors investigated include: (a) the dose and route of injection of tumour cells, (b) the effect of *Corynebacterium parvum* (*C. parvum*) and other related organisms and (c) the effect of immunosuppressive procedures such as T cell depletion and cyclophosphamide administration. In addition, we have performed investigations to establish whether Fc receptor bearing cells are present in tumours arising following the transplantation of tumour cell suspensions devoid of Fc positive cells. Finally, we have also assessed the phagocytic activity of the Fc receptor bearing cells present in our tumour.

### MATERIALS AND METHODS

*Mice.*—The experiments were performed in inbred CBA mice (both male and female) aged 8–12 weeks. These mice were bred by brother–sister mating from mice obtained from the M.R.C. Laboratory Animals Centre, Carshalton, Surrey.

*Tumours.*—The tumour used was an isogeneic methylcholanthrene induced fibrosarcoma in its 18th transplant generation. Details of the induction and propagation of this tumour are recorded elsewhere (Woodruff and Boak, 1966). Tumour cell suspensions were prepared from freshly excised tumours by teasing a tumour apart in 5–10 ml of a pronase Dulbecco solution and then incubating the suspension obtained for 5–10 min at 37°C in the same solution. The pronase Dulbecco solution contained 2·5 g of grade B pronase (Calbiochem Ltd, London, England), $5 \times 10^5$ u penicillin, 5 mg streptomycin and 0·2 mg neomycin in one litre of Dulbecco balanced salt solution (Oxoid, London, England) adjusted to pH 7·2. A short period of incubation was adopted to avoid possible changes in the tumour cell surface. The cell suspension obtained was washed 3 times in Minimal Essential Medium (MEM) (Gibco-Biocult Glasgow, Scotland) and adjusted to $2 \times 10^6$ cells/ml before transplantation or use in the rosette assay. Unless otherwise stated, the cells were transplanted by subcutaneous injection into the right thigh. The growth of the tumour was assessed continuously throughout each experiment by measuring 2 diameters at right angles. At the end of the experiment the mice were killed by ether inhalation or cervical dislocation.

*Bacterial organisms.*—The formalin killed suspension of *C. parvum* strain no. CN6134 was a gift from the Wellcome Research Laboratories, Beckenham, Kent. Formalin killed suspensions of the other organisms used, namely *Propionibacterium freudenreichii* strain no. 10470 and *C. parvum* strain no. 10387, were kindly provided by Dr W. H. McBride of the Department of Bacteriology, University of Edinburgh. They had been prepared as previously described from organisms obtained from the National Collection of Type Cultures, Colindale, England (McBride *et al.*, 1975). Details of the dose and route of injection of these suspensions is contained in the Tables. For practical reasons, smaller doses of *C. parvum*

were used when it was injected subcutaneously (see Table V) or immediately adjacent to the site of a tumour (see Table VI). Unless otherwise stated, strain CN6134 was used throughout these studies.

*Rosette technique.*—The proportion of Fc receptor bearing cells in tumour cell suspensions was assessed by determining the ability of such cells to form rosettes with antibody coated sheep erythrocytes (SRBC). The rosetting technique was undertaken as follows:

Antiserum for coating the SRBC was produced in CBA mice by injecting them intraperitoneally (i.p.) with $4 \times 10^8$ washed SRBC and bleeding them out 10 days later. It was inactivated at 56°C for 30 min before use in the rosette assays. Studies undertaken with Sephadex G-200 fractions of this serum indicated that the maximum number of rosettes was obtained with SRBC sensitized with the 7S fraction (see also Milgrom *et al.*, 1968). A series of preliminary experiments was also performed to ascertain the optimum conditions for the rosette forming assay. On the basis of the results obtained (see Fig. 1) the following procedure was adopted:

Sensitized SRBC (EA-SRBC) were prepared by incubating 1 ml of washed 5% (vol/vol) SRBC with 1 ml of a 100-fold dilution of mouse anti SRBC for 30 min at 37°C. The sensitized cells were then washed 3 times and resuspended in 5 ml of the MEM used throughout the sensitization procedure.

The percentage of rosette forming cells in a tumour was determined by mixing 0·25 ml of tumour cell suspension (containing $2 \times 10^6$ cells/ml) with 0·25 ml of 1% EA-SRBC. This mixture was then centrifuged for 5 min at 2000 rev/min, after which it was incubated at 37°C for 30 min. Following incubation, the cells were carefully resuspended using a Pasteur pipette and the percentage of rosette forming cells determined microscopically by counting at least 200 tumour cells. In all cases the rosette forming tumour cells were readily discernible (see Fig. 2), cells with more than 10 adherent EA-SRBC being regarded as positive.

The number of Fc receptor bearing cells (that is, rosette forming cells) capable of phagocytosing EA-SRBC was determined by preparing a tumour cell EA-SRBC mixture

FIG. 1—Factors influencing the sensitivity of the rosetting technique used to detect Fc receptors upon tumour cells.

The number of rosette forming cells was determined after the mixture of tumour cells and antibody coated SRBC was subjected to the conditions indicated in the figure. On the basis of these results the SRBC were sensitized with antibody diluted 100-fold and the rosettes were routinely counted after centrifugation and incubation at 37°C for 30 min.

as above, adding 50$\mu$l of heat inactivated foetal calf serum and incubating for 2 h at 37°C in a shaking water bath. After incubation, the cell mixture was gently resuspended with a Pasteur pipette and 25–50 $\mu$l of the suspension was applied to a microscope slide and covered with a glass cover slip. Upon microscopic examination, 3 populations of cells could be readily distinguished, namely, (1) a population of rosette forming cells which did not exhibit phagocytosis (see Fig. 3); (2) rosette forming cells showing clear erythrophagocytosis (see Fig. 4), and (3) cells which did not bind or phagocytose EA-SRBC (see Fig. 4). The latter were designated Fc —ve cells. Preliminary studies indicated that the phagocytic Fc receptor bearing cells could be readily distinguished using SRBC sensitized with a 100-fold dilution of anti-SRBC antibody (see Fig. 5). This dilution was therefore used in all subsequent tests.

FIG. 2—The identification of Fc receptor bearing (rosette forming) cells in tumour cell suspensions. A. Rosette formed between an Fc receptor bearing cells from a transplanted MC induced fibrosarcoma and antibody coated SRBC. B. A non-receptor bearing cell from the same tumour. C. Unbound antibody coated SRBC. Phase contrast. × 450.

Additional studies also revealed that the number of cells phagocytosing antibody coated SRBC was largely unaffected by suspending the rosetting cells in tris buffered $NH_4Cl$, thus confirming that the SRBC were indeed phagocytosed and not simply adherent to the tumour cell surface. Parallel studies also indicated that the cells which phagocytosed antibody coated SRBC also took up colloidal carbon, thus confirming their phagocytic properties. It should also be stressed that the rosette formation observed could be blocked by pretreating the tumour cell suspension with heat aggregated IgG. This treatment is used routinely to block the formation of rosettes between EA-SRBC and B cells or monocytes. In order to compensate for possible variations in the sensitivity and reproductivity of the rosetting technique, untreated tumour bearing control groups were included in every experiment when effects of therapy were being investigated. In addition, with one unavoidable exception (see Table III) the assays in all groups were always performed in parallel.

*Cell separation procedures.*—Preparations rich in Fc receptor bearing cells were isolated from tumour cell suspensions by the following procedure: Tumour cell suspensions prepared as described earlier were resuspended at a concentration of $2\text{–}3 \times 10^6$ cells/ml in RPMI 1640 medium (Gibco-Biocult, Glasgow, Scotland) containing 10% (vol/vol) heat inactivated foetal calf serum, 2 mmol/l glutamine, 100 u penicillin/ml and 100 $\mu$g streptomycin/ml. Four ml of this suspension was poured into a 5 cm tissue culture Petri dish (Product No 302V supplied by Sterilin Ltd, Richmond, Surrey, England). The dish was sealed with parafilm and incubated for 2 h at 37 °C. At the end of this time the non-adherent cells were carefully removed by decantation and the

FIG. 3.—The identification of non-phagocytic Fc receptor bearing cells in tumour cell suspensions. Note the multilayered nature of the rosette and the absence of any phagocytosis. The multilayered nature of the rosettes in this and subsequent figures is due either to (a) the cross-linking of erythrocytes by free combining sites on some of the anti-SRBC antibody molecules used to coat the SRBC or (b) the simple displacement of loosely bound red cells from the surface of the tumour cell as a result of applying the pressure of the cover slip. Oil immersion with bright field illumination. × 915.

adherent cells gently washed 3 times with RPMI-FCS, the washings being decanted on each occasion. Finally the adherent cells were dislodged from the dish by rubbing with a rubber policeman. Practically all of the adherent cells recovered formed rosettes with EA-SRBC.

Tumour cell suspensions deficient in Fc receptor bearing cells were obtained by 2 procedures. The first involved the further processing of the non-adherent cell population removed above. These cells were incubated for a second and third time as described in the preceding paragraph. The non-adherent cell population obtained after the third incubation was totally devoid of Fc receptor bearing cells. The second approach involved culturing tumour cell suspensions in glass bottles for 10 days as previously described by Ghaffar *et al.* (1974). These cells were subcultured at least 4 times before harvesting.

*Presentation of results.*—The tumour diameter values recorded are geometric mean values together with the limits of one standard error from the mean. The Fc receptor bearing cells (both phagocytic and non-phagocytic) and the Fc negative cells have been calculated as a percentage of the total number of cells in the original tumour cell suspension and the values recorded are arithmetic mean values ± the s.e. mean. The significance of the results has been assessed using the Student *t*-test procedure, values of $P \leqslant 0{\cdot}05$ being regarded as significant.

FIG. 4.—The identification of phagocytic Fc receptor bearing cells and non- receptor bearing cells in tumour cell suspensions.
Note the phagocytic Fc receptor bearing cell (A) and the non-receptor bearing cells (B). Oil immersion with bright field illumination. × 365.

### RESULTS

### *The effect of tumour cell dose, route of injection and time on the incidence of Fc receptor bearing cells in a transplanted syngeneic fibrosarcoma*

The effect of cell dose was investigated by injecting mice s.c. with between $1 \times 10^3$ and $1 \times 10^6$ viable tumour cells, killing the mice 16 days later and then determining both the phagocytic and non-phagocytic Fc receptor bearing cell content of individual tumour cell suspensions. The results of this experiment are summarized in Table I.

It will be observed that the total Fc receptor bearing cell content was inversely proportional to the size of the initial tumour inoculum. Furthermore, the higher incidence of Fc receptor bearing cells in mice challenged with the lower tumour doses was due almost entirely to an increase in the phagocytic component.

The influence of the route of transplantation of tumour cells on the Fc receptor cell content of the resultant tumour was assessed by challenging mice with $1 \times 10^6$ viable tumour cells by the i.d., s.c., i.m. or i.p. routes and determining the Fc receptor bearing cell content of the excised tumour 14 days later. From the results summarized in Table II it is apparent that both the phagocytic and non-phagocytic Fc receptor bearing cell contents of tumours are not greatly influenced by the route of tumour cell transplantation.

FIG. 5.—The detection of phagocytic and non- phagocytic cells in tumour cell suspensions using SRBC sensitized with a range of dilutions of antibody.

On the basis of the results shown the phagocytic and non-phagocytic Fc receptor bearing cell content of tumour cell suspensions were routinely investigated using SRBC sensitized with a 100-fold dilution of antibody.

An examination of the total Fc receptor bearing cell content of tumours removed at different times following transplantation indicated that as time progressed, and the tumour increased in size, so the percentage of Fc receptor bearing cells decreased (see Table III).

*The influence of bacterial organisms on the Fc receptor bearing cell content of tumours*

In view of the observations that *C. parvum* and other bacterial organisms readily inhibit the growth of the MC fibrosarcoma used in these studies (McBride *et al.*, 1975), a number of experiments were performed to ascertain if administration of these organisms influenced the Fc receptor bearing cell content of tumours. While initially we hoped that studies along these lines might throw further light on the mechanism whereby adjuvants inhibit tumour growth, the somewhat inconsistent effects of *C. parvum* (see Tables IV–VII) confused rather than clarified the situation.

In our initial studies mice were challenged with $1 \times 10^5$ or $1 \times 10^6$ tumour

TABLE I.—*The Effect of the Dose of Tumour Cells Injected on the Incidence of Fc Receptor Bearing Cells in a Transplanted Syngeneic MC Fibrosarcoma**

| | | | | % Tumour cells‡ | | |
|---|---|---|---|---|---|---|
| Group | Nos. of mice | Nos. of tumour cells injected s.c. | Tumour diameter (mm)† | With Fc receptors but non-phagocytic | With Fc receptors and phagocytic | Without Fc receptors |
| A | 4 | $1\times10^3$ | 7·2 (6·4–8·1) | 19·4±2·6 | 44·3±4·7 | 36·3±3·9 |
| B | 8 | $1\times10^4$ | 10·6 (9·6–11·7) | 23·0±3·2 | 32·5±1·9§ | 43·9±3·3 |
| C | 8 | $1\times10^5$ | 15·6 (14·8–16·5) | 25·5±3·6 | 31·2±3·9§ | 41·4±4·6 |
| D | 8 | $1\times10^6$ | 19·6 (18·9–20·3) | 17·3±4·1 | 20·2±1·8§ | 63·8±4·7‖ |

* All measurements made 16 days after tumour transplantation.
† Geometric mean with the limits of one standard error from the mean.
‡ Arithmetic mean ± s.e.
§ Significantly lower than in Group A (*P* 0·02–0·001).
‖ Significantly greater than in Group A ($P < 0·005$).

*Note.*—The phagocytic Fc receptor bearing cell content of the tumour is inversely proportional to the dose of tumour cells initially transplanted while the percentage of cells without Fc receptors is directly proportional.

TABLE II.—*The Effect of the Route of Injection of Tumour Cells on the Incidence of Fc Receptor Bearing Cells in a Transplanted Syngeneic MC Fibrosarcoma**

| Group | Nos. of mice | $1\times10^6$ tumour cells injected | Tumour diameter† (mm) | % Tumour cells‡: With Fc receptors but non-phagocytic | With Fc receptors and phagocytic | Without Fc receptors |
|---|---|---|---|---|---|---|
| A | 6 | Intradermally (i.d.) | 9·3 (9·2–9·5) | 24·7±11·6 | 35·7±2·9 | 39·2±5·2 |
| B | 6 | Subcutaneously (s.c.) | 11·7 (11·1–12·4) | 27·0±3·5 | 36·2±1·8 | 40·6±4·3 |
| C | 6 | Intramuscularly (i.m.) | 12·8 (12·2–13·5) | 20·6±6·3 | 43·8±5·4 | 35·6±5·9 |
| D | 3 | Intraperitoneally (i.p.) | | 28·3±5·0 | 29·3±2·7 | 42·3±3·4 |

* All measurements made 14 days after tumour transplantation.
† Geometric mean with the limits of one standard error from the mean.
‡ Arithmetic mean ± s.e.
*Note.*—The route of transplantation of the tumour cell inoculum has a negligible effect on the receptor bearing cell content of the resultant tumour.

TABLE III.—*The Effect of Time on the Incidence of Fc Receptor Bearing Cells in a Transplanted Syngeneic Fibrosarcoma**

| Group | Nos. of mice | Day examined | Tumour diameter (mm)† | % Cells with Fc receptors‡ |
|---|---|---|---|---|
| A | 5 | 12 | 14·1 (13·8–14·4) | 40·4±8·2 |
| B | 5 | 14 | 16·8 (16·2–17·4) | 32·8±6·8 |
| C | 7 | 18 | 22·2 (21·9–22·5) | 16·7±3·2§ |

* 1 × $10^6$ tumour cells injected s.c. on Day 0.
† Geometric mean with the limits of one standard error from the mean.
‡ Arithmetic mean ± s.e.
§ Significantly lower than in Group A or Group B ($P$ 0·005–<0·001).
*Note.*—The total Fc receptor bearing cell content of the tumour declines as its age (and size) increases.

TABLE IV.—*The Effect of* C. parvum *on the Incidence of Fc Receptor Bearing Cells in a Transplanted Syngeneic MC Fibrosarcoma*

| Group | Nos. of tumour cells transplanted | *C. parvum* administered (1·4 mg i.p.) | Tumour diameter (mm)*: Day 12 or 13 | Day 28 or 29 | % Cells with Fc receptors†: Day 12 or 13 | Day 28 or 29 |
|---|---|---|---|---|---|---|
| A | $1\times10^5$ | No | 8·4 (7·7–9·3) | 9·7‡ (9·5–10·0) | 46·0±10·4 | 36·5±0·7‡ |
| B | $1\times10^5$ | Yes | 6·0 (5·6–6·4) | 7·2 (6·4–8·1) | 56·0±8·3 | 67·3±28·3§ |
| C | $1\times10^6$ | No | 10·7 (9·6–11·9) | 16·0 (12·6–20·5) | 49·3±7·1 | 31·5±10·3 |
| D | $1\times10^6$ | Yes | 8·9 (8·2–7·8) | 14·0 (12·0–16·4) | 48·0±6·6 | 45·8±11·8§ |

* Geometric mean together with the limits of one standard error from the mean.
† Arithmetic mean ± s.e.
‡ Only 2 mice examined. On all other occasions a minimum of 3 examined.
§ Significantly greater than in non-*C. parvum* treated controls.
*Note.*—The administration of *C. parvum* appears to halt the decline in the relative proportion of Fc receptor bearing tumour cells.

TABLE V.—*The Effect of the Route of Administration of* C. parvum *on the Incidence of Fc Receptor Bearing Cells in a Transplanted Syngeneic MC Fibrosarcoma**

| Group | Nos. of mice | Treatment | Tumour diameter (mm)† | % cells with Fc receptors‡ |
|---|---|---|---|---|
| A | 10 | None | 11·2 (9·8–12·8) | 45·1±8·4 |
| B | 10 | *C. parvum* (0·7 mg s.c. Day 3) | 10·6 (9·8–11·4) | 41·0±6·6 |
| C | 7 | *C. parvum* (0·7 mg i.p. Day 3) | 8·0 (7·2–8·9) | 43·9±4·3 |

* All animals injected s.c. with 1 × $10^5$ tumour cells on Day 0 and assays performed 23 days later.
† Geometric mean together with the limits of one standard error from the mean.
‡ Arithmetic mean ± s.e.
*Note.*—On this occasion the administration of *C. parvum* had no effect on the overall proportion of Fc receptor bearing cells.

TABLE VI.—*The Effect on the Incidence of Fc Receptor Bearing Cells in a Transplanted Syngeneic MC Fibrosarcoma of* C. parvum *Administered Adjacent to the Tumour Site*

| | | | | | % Tumour cells‡ | | |
|---|---|---|---|---|---|---|---|
| Group | Nos. of mice | Day examined | Treatment* | Tumour diameter (mm)† | With Fc receptors but non-phagocytic | With Fc receptors and phagocytic | Without Fc receptors |
| A | 11 | 16 | Nil | 18·8 (18·4–19·3) | 15·9±1·8 | 26·6±2·1 | 57·5±2·5 |
| B | 6 | 16 | 0·7 mg *C. parvum*§ injected adjacent to tumour on Day 8 | 18·9 (18·2–19·6) | 23·4±2·5‖ | 31·0±2·0 | 45·7±6·8‖ |
| C | 4 | 21 | Nil | 24·1 (23·8–24·4) | 36·5±7·7 | 25·2±5·9 | 38·3±5·3 |
| D | 4 | 21 | 0·7 mg *C parvum* injected adjacent to tumour on Day 8 | 21·9 (21·0–22·8) | 28·8±3·6 | 30·8±6·0 | 40·5±7·9 |

* All mice injected s.c. with 1 × $10^6$ tumour cells on Day 0.
† Geometric mean with the limits of one standard error from the mean.
‡ Arithmetic mean ± s.e.
§ At the time of *C. parvum* injection tumour diameter was 9 mm and the total Fc receptor bearing cell content of tumour excised at this time was 64%.
‖ Significantly different from control group A (*P* 0·01–0·025).
*Note.*—In general, the administration of *C. parvum* close to the site of a growing tumour had little effect on the Fc receptor bearing cell content of the tumour.

TABLE VII.—*The Effect of Various Bacterial Organisms on the Incidence of Fc Receptor Bearing Cells in a Transplanted Syngeneic MC Fibrosarcoma*

| Group | Nos. of mice | Organism injected* | Tumour diameter (mm)† | % Cells with Fc receptors‡ |
|---|---|---|---|---|
| A | 8 | None | 16·3 (15·5–17·2) | 39·7±7·1 |
| B | 8 | *C. parvum* strain No. CN6134 | 9·2§ (7·9–10·3) | 56·3±10·0§ |
| C | 8 | *Propionibacterium freudenreichii* strain No. 10470 | 14·6 (13·3–16·1) | 35·8±10·3 |
| D | 8 | *C. parvum* strain No. 10387 | 9·0§ (8·1–9·9) | 58·4±8·6§ |

* The organisms were injected i.p. 3 days after the transplantation of 1 × $10^5$ tumour cells. In Groups B and C 1·4 mg dry weight of organism was injected, while in Group D, 0·7 mg.
† Geometric mean together with the limits of one standard error from the mean.
‡ Arithmetic mean ± s.e.
§ Significantly different from untreated controls (*P* 0·005–<0·001).
*Note.*—On this occasion the preparations which inhibit tumour growth also result in a marked increase in the Fc receptor bearing cell content of the tumour.

cells and injected i.p. 3 days later with 1·4 mg of *C. parvum*. Control mice received tumour alone. These studies suggested (see Table IV) that the *C. parvum* protocol used did not exert a dramatic effect upon the Fc receptor bearing cell content of transplanted tumours, at least in their early stages of growth. It did appear, however, to halt the dramatic decline in the Fc positive cell content noted in older tumours, though further experiments with larger numbers of animals will be necessary to establish this point.

An experiment was also performed to see if the effect, if any, of *C. parvum* on the Fc receptor bearing cell content of tumours was dependent upon the route of administration of *C. parvum*, as this is known to influence the antitumour effect of this reagent (Woodruff, McBride and Dunbar, 1974; Scott, 1974). In these experiments mice were challenged with tumour cells on Day 0 and injected s.c. or i.p. with 0·7 mg of *C. parvum* 3 days later. The animals were killed 23 days after tumour cell transplantation and the total Fc receptor bearing cell content of the tumours determined. It was found that the administration of *C. parvum* by either the s.c. or the i.p. route failed to influence the total Fc receptor bearing cell content of the tumours (see Table V). It should also be noted that, as previously observed, the i.p. administration of *C. parvum* significantly inhibited tumour growth while s.c. administration at a site distant from the tumour was without effect (Woodruff *et al.*, 1974; Scott, 1974).

The effect of local administration of *C. parvum* was determined by injecting 0·7 mg dry weight of this organism s.c. immediately adjacent to the site of a growing tumour. The *C. parvum* was injected 8 days after the transplantation s.c. of $1 \times 10^6$ viable tumour cells. Previous studies in our laboratory had shown that this *C. parvum* protocol inhibited the growth of tumour arising following the s.c. transplantation of $1 \times 10^4$ tumour cells (Woodruff and Dunbar, 1975). However, it will be observed that in the present experiment (see Table VI) it had little effect on tumour growth or on the Fc receptor bearing cell content of the tumour. Thus, it appears that the effectiveness of this protocol may be dependent on the number of tumour cells initially transplanted.

The most dramatic effect of *C. parvum* therapy on the total Fc positive cell content of tumours was obtained in experiments set up to compare the effects of 3 different preparations of formalin killed bacteria. In these experiments mice were challenged with $1 \times 10^5$ syngeneic tumour cells on Day 0 and 3 days later were injected i.p. with the preparations listed in Table VII. The percentage of Fc receptor bearing cells in individual tumour cell suspensions was determined 24 days after tumour transplantation. The 2 strains of *C. parvum* tested significantly inhibited tumour growth and also significantly increased the proportion of Fc receptor bearing cells in the excised tumour. In contrast, the administration of *P. freundenreichii* failed to inhibit tumour growth or to alter the incidence of Fc receptor bearing cells (see Table VII). It should be noted that the effects of *P. freundenreichii* and *C. parvum* strain 10387 on tumour growth are somewhat different than previously reported (McBride *et al.*, 1975). This might, however, be due to differences in the number of tumour cells transplanted and the weight of organisms injected.

*The incidence of Fc receptor bearing cells in immunologically deprived and immunosuppressed mice*

Previous reports from our laboratory have shown that i.p. administered *C. parvum* exerts an anti-tumour effect in T cell deprived, that is, B mice (Woodruff, Dunbar and Ghaffar, 1973). In an attempt to further elucidate the mechanism by which *C. parvum* exerts an antitumour effect in B mice the following experiment was performed:

Mice were thymectomized at the age of 4–6 weeks and one week later they received 850 rad whole body irradiation, followed 8 h later by the i.v. infusion of $4 \times 10^6$ anti-$\theta$ antibody treated isogeneic bone marrow cells. Six weeks after reconstitution the mice were challenged with $1 \times 10^4$ tumour cells and after a further interval of 3 days 1·4 mg of *C. parvum* was injected i.p. Control mice were sham thymectomized, irradiated and reconstituted as above.

The incidence of Fc positive tumour cells was no different in thymectomized mice than in sham thymectomized controls (see Groups A and C, Table VIII). Furthermore, the percentage of such cells was not significantly affected by the administration of *C. parvum* even though it significantly inhibited the growth of tumour in the B mice. It will be noted, however, that the proportion of Fc positive cells in all groups was greater than normally observed. Whether or not this is a property of tumours grown in irradiated bone marrow reconstituted mice, or a reflection of the variability of the rosetting procedure, remains to be established.

The effect of immunosuppressive treatment on the Fc receptor bearing cell content of tumours was investigated by treating tumour challenged mice with cyclophosphamide as indicated in Table IX. This treatment was found to significantly inhibit the growth of transplanted tumour and at the same time significantly increase its proportion of phagocytic Fc receptor bearing cells. The proportion of non-phagocytic Fc positive cells was not significantly affected by cyclophosphamide administration.

*Fc receptor bearing cells in tumours grown from Fc deficient and Fc enriched tumour cell suspensions*

In these experiments mice were challenged s.c. with tumour cell suspensions deficient or enriched in Fc receptor bearing cells. These suspensions were prepared as described earlier. Both types of preparation gave rise to tumours in which there were cells expressing Fc receptors on their surface (see Table X). The Fc receptor bearing cells were found in all the tumours grown from Fc negative tumour cell suspensions and were present in high numbers within 7 days of tumour cell transplantation.

### DISCUSSION

These studies demonstrate convincingly that a significant proportion of the

TABLE VIII.—*The Incidence of Fc Receptor Bearing Tumour Cells in B Mice and the Effect of* C. parvum *Therapy Thereupon*

| Group | Nos. of mice | Treatment | Tumour diameter (mm)* | % Cells with Fc receptors† |
|---|---|---|---|---|
| A | 4 | Sham thymectomized mice, x-irradiated, repopulated with anti-$\theta$ treated bone marrow cells and challenged (s.c.) with $1 \times 10^4$ tumour cells | 12·1 (10·5–14·0) | 84·8±8·3 |
| B | 4 | As in A but also injected (i.p.) with 1·4 mg *C. parvum* 3 days after tumour transplantation | 8·2 (7·1–9·5) | 62·3±14·1 |
| C | 7 | Thymectomized mice, x-irradiated, repopulated with anti-$\theta$ treated bone marrow cells and challenged (s.c.) with $1 \times 10^4$ tumour cells | 14·4 (13·7–15·2) | 75·6±2·2 |
| D | 8 | As in C but also injected (i.p.) with 1·4 mg *C. parvum* 3 days after tumour transplantation | 11·2‡ (10·7–11·9) | 79·0±5·4 |

* Geometric mean together with the limits of one standard error from the mean.
† Arithmetic mean ± s.e.
‡ Significantly lower than in Group C ($P \simeq 0\cdot005$).
*Note* the high incidence of Fc receptor bearing cells in tumours grown in B mice. In addition, this is unaffected by *C. parvum* administration.

TABLE IX.—*The Effect of Cyclophosphamide on the Incidence of Fc Receptor Bearing Cells in a Transplanted Syngeneic MC Fibrosarcoma**

| Group | Nos. of mice | Treatment | Tumour diameter (mm)† | % Tumour cells‡: With Fc receptors but non-phagocytic | With Fc receptors and phagocytic | Without Fc receptors |
|---|---|---|---|---|---|---|
| A | 11 | 1 × $10^6$ tumour cells (s.c.) on Day 0 | 18·8 (18·4–19·3) | 15·9±1·8 | 26·6±2·1 | 57·5±2·3 |
| B | 8 | 1 × $10^6$ tumour cells (s.c.) on Day 0 and cyclophosphamide (200 mg/kg i.p.) on Days −5 and 8 | 9·7 (9·0–10·5) | 21·6±2·7 | 43·8±2·3§ | 34·6±3·4‖ |
| C | 8 | 1 × $10^6$ tumour cells (s.c.) on Day 0 and cyclophosphamide (200 mg/kg i.p.) on Day 8 | 10·5 (9·5–11·6) | 12·3±2·8 | 41·0±3·9§ | 46·8±5·0‖ |

* All assays performed 16 days after tumour transplantation.
† Geometric mean values together with the limits of one standard error from the mean.
‡ Arithmetic mean ± s.e.
§ Significantly higher than in non-cyclophosphamide treated controls ($P$ 0·005–<0·001).
‖ Significantly lower than in non-cyclophosphamide treated controls ($P$ 0·005–<0·001).
*Note* the increased percentage of phagocytic Fc receptor bearing cells in tumours from cyclophosphamide treated mice.

TABLE X.—*The Incidence of Fc Receptor Bearing Cells in Tumours Grown from Fc Deficient and Fc Enriched Tumour Cell Suspensions*

| Group | Tumour cells transplanted*: Source | % with Fc receptors | Characteristics of resultant tumour† | Day measured: 7 | 13 | 16 | 18 | 28 |
|---|---|---|---|---|---|---|---|---|
| A | Cultured tumour cells | 0 | % cells with Fc receptors | | | 58 | 45, 52, 57, 60 | 38 |
| | | | Tumour diameter (mm) | | | 7·5 | 9, 13, 8·5 | 22 |
| B | Non-adherent cells from freshly excised tumour | 0 | % cells with Fc receptors | 65 | 36, 58, 57, 75 | 36 | | |
| | | | Tumour diameter (mm) | 6·5 | 12, 11, 10, 5 | 12 | | |
| C | Adherent cells from freshly excised tumour | 99·9 | % cells with Fc receptors | 75 | 85, 98 | 60 | | 51, 99 |
| | | | Tumour diameter (mm) | 3 | 5, 8 | 6 | | 19, 20 |

* 1 × $10^5$ cells transplanted s.c.
† The values expressed are for individual mice.
*Note.*—Tumour cell suspensions devoid of Fc receptor bearing cells gave rise to tumours containing a high proportion of Fc receptor bearing cells.

cells in our transplanted MC induced fibrosarcoma have Fc receptors on their surface for they readily form rosettes with antibody coated SRBC, thus confirming the results noted with a variety of other tumours (Milgrom *et al.*, 1968; Cohen *et al.*, 1971; Tønder *et al.*, 1974; Kerbel and Davies, 1974). They also extend previous observations by throwing further light on the nature of the Fc receptor bearing cells and factors which influence their incidence in tumours. They fail, however, to establish their relationship, if any, to tumour growth.

A number of observations lead us to believe that many (though not all) of the Fc receptor bearing cells in our tumour are probably infiltrating macrophages of host origin. Such a possibility had previously been suggested by Kerbel and Davies (1974) on the basis of the observations that certain tumours contain large numbers of infiltrating macrophages (Evans, 1972), and these cells are known to possess Fc receptors on their surface (Lay and Nussensweig, 1968). The Fc receptor bearing cells in our tumour adhere to plastic and glass and many of them exhibit phagocytosis of antibody coated SRBC. In addition, other observations indicate that a large proportion of these cells phagocytose colloidal carbon (L. Gruer, unpublished) and preliminary studies suggest that they can be separated by a magnet following ingestion of carbonyl iron. The rapid appearance of Fc receptor bearing cells in tumours grown from Fc negative tumour cell suspensions leads one to conclude that the Fc positive cells are most probably infiltrating cells of host origin, a conclusion also reached by Evans (1972) with respect to host macrophages. However, before the host origin of these cells can be established beyond all reasonable doubt, further studies will be necessary with tumours transplanted in $F_1$ hybrid mice or CBA T6 mice. Such studies would permit recognition of host cells by their cell surface antigens or chromosome markers.

Although all Fc receptor bearing cells can be removed by incubation in plastic Petri dishes, or by subculture of cell lines in glass bottles, a significant proportion of these cells do not phagocytose antibody coated SRBC or colloidal carbon. Whether or not these non-phagocytic cells are " inactive macrophages ", genuine tumour cells or other lymphoreticular cells still remains to be established. While the observation (see Table X) that tumours grown from cell preparations consisting almost entirely of Fc receptor bearing cells could be taken to indicate that tumour cells as such may express Fc receptors on their surface, the possibility still remains that the tumours arose from small numbers of Fc negative tumour cells present within the inoculum. The transplantation of a range of doses of the Fc enriched suspension should help resolve this matter. Other studies currently under way in our laboratory indicate that our tumour cell suspensions also lyse antibody coated erythrocytes in culture, that is, exert K cell cytolysis (Ghaffar *et al.*, 1975). Experiments are in progress to characterize the effector cell in this system and to establish if it is of host origin.

The present studies demonstrate that a number of factors influence the relative proportion of Fc receptor bearing cells in our tumour. For example, it may be increased by transplanting small doses of tumour cells (contrast Evans, 1972), by the administration of cyclophosphamide and on occasion by the i.p. injection of certain adjuvants. In all these cases it is interesting to note that the procedures used limit the size of the tumour. Conversely, if large tumour doses are transplanted, or the tumour is allowed to grow for a longer time, then the proportion of Fc receptor bearing cells decreases. This is undoubtedly due to a disproportionate increase in the Fc negative population and not to an actual decline in the absolute numbers of Fc receptor bearing cells. These observations suggest that procedures which limit tumour growth are those which favour a high Fc +ve/Fc −ve (and presumably macrophage/tumour) cell ratio. If this is so, then one has to explain the somewhat inconsistent effects of *C. parvum* for occasionally it exerted an appreciable antitumour effect without increasing the proportion of Fc receptor bearing cells (see Table V). In addition, Eccles and Alexander (1974) have shown that BCG can exert an anti-tumour effect without significantly increasing the macrophage content of tumours. Nevertheless, it is conceivable that the *C. parvum* protocol used in Table V increased the proportion of phagocytic Fc receptor bearing cells and that this population influences tumour growth.

The observation that tumours grown in B mice also have as many Fc receptors on their cell surfaces as appropriate control mice is of interest. As expected, it confirms that these adherent Fc receptor bearing cells are not of T origin or dependent upon T cells for their generation. Furthermore, it also suggests that their localization within tumours is not a consequence of the release of soluble factors (lymphokines) following contact of tumour antigen and T cells. In contrast to our observations others have reported that prior thymectomy and x-irradiation of rats influences the macrophage content of a transplanted syngeneic benzpyrene induced fibrosarcoma, the level being much lower than in sham thymectomized controls (Eccles and Alexander, 1974). However, in these experiments the thymectomized x-irradiated rats were not repopulated with bone marrow cells as in our studies, and this may have contributed to the difference.

Although these results establish clearly that many of the cells in tumour cell suspensions bear Fc receptors upon their surface and indicate that a large proportion of such cells are phagocytic, the precise significance of these cells in relation to tumour growth remains to be established. Further studies on the proportion of phagocytic and non-phagocytic rosette forming cells in tumours and the effect of various therapeutic procedures thereupon will undoubtedly help elucidate their importance in tumour surveillance.

The authors wish to acknowledge the generous financial support of the Cancer Research Campaign, the help of Drs A. Ghaffar and W. H. McBride, and the interest of Professor Sir Michael Woodruff. They are indebted to L. Gruer who undertook part of this work, I Milne and J. Merriman for skilled technical assistance, and to the British Council for awarding a Fellowship to Dr Szymaniec.

*Addendum.*—Since submitting this paper for publication we have noted a recent article by Kerbel, Pross and Elliot (*Int. J. Cancer*, 1975, **15**, 918) recording results similar to our own. These authors demonstrate convincingly that a variety of tumours contain Fc receptor bearing cells, the majority of which are phagocytic. These cells were also lost following culture *in vitro* and transplantation of the cultured cells also gave rise to tumours containing a high proportion of Fc receptor bearing cells. Cytotoxic studies with anti-H2 sera on tumours transplanted to $F_1$ hybrids indicated that many (if not all) of the Fc receptor bearing cells were of host origin.

### REFERENCES

COHEN, D., GURNER, B. W. & COOMBS, R. R. A. (1971) A Phenomenon resembling Opsonic Adherence shown by Disaggregated Cells of the Transmissible Venereal Tumour of the Dog. *Br. J. exp. Path.*, **52**, 447.

ECCLES, S. A. & ALEXANDER, P. (1974) Macrophage Content of Tumours in Relation to Metastatic Spread and Host Immune Reaction. *Nature, Lond.* **250**, 665.

EVANS, R. (1972) Macrophages in Syngeneic Animal Tumours. *Transplantation*, **14**, 468.

GHAFFAR, A., CULLEN, R. T., DUNBAR, N. & WOODRUFF, M. F. A. (1974) Anti-tumour Effect *in vitro* of Lymphocytes and Macrophages from Mice Treated with *Corynebacterium parvum*. *Br. J. Cancer*, **29**, 199.

GHAFFAR, A., SZYMANIEC, S. & CALDER, E. A. (1975) Antibody Dependent Cell Mediated Cytotoxic Activity in a Methylcholanthrene Induced Fibrosarcoma. In preparation.

KERBEL, R. S. & DAVIES, A. J. S. (1974) The Possible Biological Significance of Fc Receptors on Mammalian Lymphocytes and Tumour Cells. *Cell*, **3**, 105.

LAY, W. H. & NUSSENSWEIG, V. (1968) Receptors for Complement on Leukocytes. *J. exp. Med.*, **128**, 991.

McBRIDE, W. H., DAWES, J., DUNBAR, N., GHAFFAR, A. & WOODRUFF, M. F. A. (1975) A Comparative Study of Anaerobic Coryneforms. Attempts to Correlate their Anti-tumour Activity with their Serological Properties and Ability to Stimulate the Lymphoreticular System. *Immunology*, **28**, 49.

MACLENNAN, I. C. M. (1972) Antibody in the Induction and Inhibition of Lymphocyte Cytotoxicity. *Transplantn Rev.*, **13**, 67.

MILGROM, F., HUMPHREY, L. J., TØNDER, O., YASUDA, J. & WITEBSKY, E. (1968) Antibody Mediated Haemadsorption by Tumour Tissue. *Int. Archs Allergy*, **33**, 478.

SCOTT, M. T. (1974) *Corynebacterium parvum* as an Immunotherapeutic Anticancer Agent. *Semin. Oncol.*, **1**, 367.

TØNDER, O., MORSE, P. A. & HUMPHREY, L. J. (1974) Similarities of Fc Receptors in Human Malignant Tissue and Normal Lymphoid Tissue. *J. Immun.*, **113**, 1162.

WOODRUFF, M. F. A. & BOAK, J. L. (1966) Inhibitory Effect of Injection of *Corynebacterium*

*parvum* on the Growth of Tumour Transplants in Isogeneic Hosts. *Br. J. Cancer*, **20**, 345.
WOODRUFF, M. F. A. & DUNBAR, N. (1975) Effect of Local Injection of *Corynebacterium parvum* on the Growth of a Murine Fibrosarcoma. *Br. J. Cancer*, **32**, 34.
WOODRUFF, M. F. A., DUNBAR, N. & GHAFFAR, A. (1973) The Growth of Tumours in T Cell Deprived Mice and their Response to Treatment with *Corynebacterium parvum*. *Proc. R. Soc. B.*, **184**, 97.
WOODRUFF, M. F. A., McBRIDE, W. H. & DUNBAR, N. (1974) Tumour Growth, Phagocytic Activity and Antibody Response in *Corynebacterium parvum*-treated Mice. *Clin. & exp. Immunol.*, **17**, 509.